# Comparative genomics between *Trichomonas tenax* and *Trichomonas vaginalis*: CAZymes and candidate virulence factors

**DOI:** 10.3389/fmicb.2024.1437572

**Published:** 2024-07-17

**Authors:** Lenshina A. Mpeyako, Adam J. Hart, Nicholas P. Bailey, Jane M. Carlton, Bernard Henrissat, Steven A. Sullivan, Robert P. Hirt

**Affiliations:** ^1^Biosciences Institute, Newcastle University, Newcastle upon Tyne, United Kingdom; ^2^Department of Biology, Center for Genomics and Systems Biology, New York University, New York, NY, United States; ^3^Department of Molecular Microbiology and Immunology, Bloomberg School of Public Health, Johns Hopkins University, Baltimore, MD, United States; ^4^Department of Biological Sciences, King Abdulaziz University, Jeddah, Saudi Arabia; ^5^Department of Biotechnology and Biomedicine (DTU Bioengineering), Technical University of Denmark, Lyngby, Denmark

**Keywords:** protein-coding genes, surface proteins, secreted proteins, glycoside/glycosyl hydrolases (GHs), peptidases/proteases, exosomes, pore-forming proteins

## Abstract

**Introduction:**

The oral trichomonad *Trichomonas tenax* is increasingly appreciated as a likely contributor to periodontitis, a chronic inflammatory disease induced by dysbiotic microbiota, in humans and domestic animals and is strongly associated with its worst prognosis. Our current understanding of the molecular basis of *T. tenax* interactions with host cells and the microbiota of the oral cavity are still rather limited. One laboratory strain of T. tenax (Hs-4:NIH/ATCC 30207) can be grown axenically and two draft genome assemblies have been published for that strain, although the structural and functional annotation of these genomes is not available.

**Methods:**

GenSAS and Galaxy were used to annotate two publicly available draft genomes for *T. tenax*, with a focus on protein-coding genes. A custom pipeline was used to annotate the CAZymes for *T. tenax* and the human sexually transmitted parasite *Trichomonas vaginalis*, the most well-characterized trichomonad. A combination of bioinformatics analyses was used to screen for homologs of *T. vaginalis* virulence and colonization factors within the *T. tenax* annotated proteins.

**Results:**

Our annotation of the two *T. tenax* draft genome sequences and their comparison with *T. vaginalis* proteins provide evidence for several candidate virulence factors. These include candidate surface proteins, secreted proteins and enzymes mediating potential interactions with host cells and/or members of the oral microbiota. The CAZymes annotation identified a broad range of glycoside hydrolase (GH) families, with the majority of these being shared between the two *Trichomonas* species.

**Discussion:**

The presence of candidate *T. tenax* virulence genes supports the hypothesis that this species is associated with periodontitis through direct and indirect mechanisms. Notably, several GH proteins could represent potential new virulence factors for both Trichomonas species. These data support a model where *T. tenax* interactions with host cells and members of the oral microbiota could synergistically contribute to the damaging inflammation characteristic of periodontitis, supporting a causal link between *T. tenax* and periodontitis.

## 1 Introduction

The trichomonad *Trichomonas tenax* (Phylum Parabasalia, Class Trichomonadea, Cepicka et al., 2010) is an obligate symbiont of the oral cavity reported to have a potential role in periodontitis (Marty et al., 2017; Santi-Rocca, 2020; Eslahi et al., 2021; Martin-Garcia et al., 2022). However, *T. tenax* is one of the least well-understood microbial species of the complex oral microbial ecosystem and very little is known on how it might modulate human oral disease (Baker et al., 2024). Similar to the closely related sexually transmitted parasite *Trichomonas vaginalis*, which is human-specific, *T. tenax* was also commonly considered to be specifically associated with humans (Honigberg, 1990; Maritz et al., 2014). However, the use of sensitive molecular diagnostic tools has clearly demonstrated that *T. tenax* is common in the oral cavity of a broader range of hosts including, cats, dogs and horses and with some additional reports among birds (Kellerová and Tachezy, 2017; Matthew et al., 2023). There are also reports of the isolation of *T. tenax* from extra-oral locations including the respiratory tract, lymph nodes, salivary glands and the urogenital tract (Maritz et al., 2014; Brosh-Nissimov et al., 2019). Studies have reported an increased prevalence of *T. tenax* among patients with periodontitis compared to healthy controls (Marty et al., 2017; Eslahi et al., 2021; Martin-Garcia et al., 2022). However, the ability of *T. tenax* to contribute, directly or indirectly, to the pathobiology of periodontitis, is still poorly understood. Indeed, there is a lack of data on the molecular and cellular basis of *T. tenax* interactions with host cells (Matthew et al., 2023), members of the microbiota, and its potential endosymbionts, three key factors considered important in modulating the pathobiology of *T. vaginalis* (Hirt et al., 2011; Mercer and Johnson, 2018; Riestra et al., 2022; Margarita et al., 2023).

Trichomonad genomes typically exceed 50 Mb, making them unusually large compared to other parasitic microbial eukaryotes (Zubáčová et al., 2008). The published draft genome data for *T. vaginalis* (G3) is consistent with these estimates, with a size of ~160 Mb (Carlton et al., 2007). The first draft whole genome sequence for *T. tenax* (strain Hs-4:NIH, ATCC 30207; Manassas, VA, USA) was published in 2019 (~47 Mb, across 4,161 contigs) (Benabdelkader et al., 2019). Benabdelkader et al. (2019) conducted this study to investigate the prevalence and genetic diversity of *T. tenax* and its potential involvement in the severity of periodontitis among humans. A second draft whole genome sequence dataset for the same strain of *T. tenax* was published in 2022 (63.4 Mb, across 4906 contigs) (Yang et al., 2022). Both draft genomes are smaller than the 133 ± 4 Mb estimated from flow cytometry (Zubáčová et al., 2008). The two published *T. tenax* draft genomes did not include annotation of protein-coding genes, which is essential to guide the study of the molecular cell biology of a given organism. Here, we investigated *T. tenax* protein-coding genes by integrating and annotating the two published draft genomes (Benabdelkader et al., 2019; Yang et al., 2022). The predicted *T. tenax* protein-coding genes and their functional annotations were compared with the predicted proteome of a new assembly of *T. vaginalis*. We also annotated the CAZymes (Lombard et al., 2014; Wardman et al., 2022) from *T. tenax* and *T. vaginalis*. We focused our analyses to specifically test the hypothesis that *T. tenax* encodes a range of virulence and colonization factors that contribute directly and indirectly to the pathobiology of periodontitis through interaction with various host cells and members of the oral microbiota, as known for *T. vaginalis* (Hirt, 2013; Mercer and Johnson, 2018; Riestra et al., 2022). Indeed, only a handful of publications have investigated the potential virulence of *T. tenax* on host cells and the virulence factors that could be associated with the observed impact on host cells (reviewed in Matthew et al., 2023). Similarly, we are aware of only one study that has considered *T. tenax* genes encoding candidate proteins mediating interactions with members of the microbiota, i.e. the *T. vaginalis* NlpC/P60 peptidases that target bacterial cell walls and are conserved in *Trichomonas gallinae* (Barnett et al., 2023). Here we took advantage of the rich annotation available for *T. vaginalis* virulence factors (Carlton et al., 2007; Hirt et al., 2011; Hirt, 2013; Mercer and Johnson, 2018; Riestra et al., 2022) to expand the bioinformatic characterization of *T. tenax* candidate virulence factors. These could mediate binding of *T. tenax* to human epithelial cells, immunocytes, the microbiota in the oral cavity, or could degrade host structural proteins or those involved in innate and adaptive immunity, and by doing so could contribute to the pathobiology of periodontitis.

## 2 Materials and methods

### 2.1 Genome annotation

GenSAS (Humann et al., 2019) was used to annotate the two draft genome sequences of *T. tenax* strain Hs-4:NIH (NCBI Genome assemblies PRJEB22701 and ASM2309173v1) (Benabdelkader et al., 2019; Yang et al., 2022). Identification and masking of repeats was achieved using RepeatMasker (Nishimura, 2000) and RepeatModeler (Smit and Hubley, 2008) on GenSAS. Multiple tools integrated into GenSAS were employed for the *ab initio* prediction of protein-coding genes including Augustus (Stanke et al., 2004), GeneMarkES (Lomsadze et al., 2005), GlimmerM (Salzberg et al., 1999), Genescan (Burge and Karlin, 1997) and SNAP (Korf, 2004). For homology-based gene predictions protein and transcript sequences from other trichomonads, including *T. vaginalis* (Carlton et al., 2007), and other relevant eukaryotic species were downloaded from NCBI and aligned to the genomes using BLAST (Altschul et al., 1990), BLAT (Kent, 2002), PASA (Haas et al., 2003) and DIAMOND (Buchfink et al., 2021) using default parameters. A published *T. tenax* RNA–Seq dataset (Handrich et al., 2019) (NCBI SRA accession SRX2052871) was used as evidence for expression of protein-coding genes. The RNA–Seq reads were aligned to the repeat masked genome assembly using HISAT2 (Kim et al., 2015) and TopHat2 (Kim et al., 2013) with default parameters. EvidenceModeler (Haas et al., 2008) was used to create the consensus gene set by incorporating the outputs of all *ab initio* and alignment-based gene predictions. The official gene set (OGS) which is defined as the set of predictions that serve as the most trusted gene predictions, was generated by PASA Refinement (Haas et al., 2003). In this study, all outputs generated from the gene prediction tools were included and integrated to generate a consensus annotation.

### 2.2 Protein functional annotation

For protein functional annotation, tools integrated into GenSAS were used and included DIAMOND (Buchfink et al., 2021) and InterProScan (Jones et al., 2014). These analyses were complemented with tools integrated into Galaxy (Afgan et al., 2016) and included BlastP (Camacho et al., 2009), EggNog Mapper (Huerta-Cepas et al., 2016) and Motif Search (Kanehisa, 2002), PANZZER2 (Törönen et al., 2018) and GhostKoala (Kanehisa et al., 2016). The predicted proteins were used to query the NCBI non-redundant (nr) refseq_protozoa protein database (released on 9/12/2021) using the BLASTP search, with an E-value cut-off of 1 × 10^−8^. That version of the database contains 1,095,419 protein sequences from different protozoans including *T. vaginalis*. The inferred protein sequences of *T. tenax* were also used to query the SWISS-PROT and TrEMBL databases using DIAMOND to further improve the accuracy of function allocation. Gene name assignment was done by recording BLASTP top hits and PANZZER2 (Törönen et al., 2018). Additional functional domains were predicted by EggNog Mapper (Huerta-Cepas et al., 2017). Default settings were used with a minimum E-value expected to be 1 × 10^−3^. Both InterProScan and Eggnog Mapper provided additional evidence for existing gene annotations including the database cross-references (Dbxref) and gene ontology terms (GO). We also used SMART (Letunic and Bork, 2018) to investigate the domain organization of specific proteins of interest. To further enrich the protein functional annotation, GhostKOALA (Kanehisa et al., 2016) was used to assign K numbers to the annotations by GHOSTX search against a nonredundant set of KEGG GENES. The GhostKOALA platform is a web-based server that performs automatic annotation of genomes with Kyoto Encyclopedia of Genes and Genomes (KEGG) orthology (Kanehisa et al., 2016). Protein localization was inferred using Phobius (Käll et al., 2007), SignalP (Petersen et al., 2011) and for selected entries DeepTMHMM (Hallgren et al., 2022). Data produced by the different software were mined and an annotation summary generated manually.

In addition to the published 2007 annotation of the *T. vaginalis* G3 draft genome (Carlton et al., 2007), we leveraged updated annotation from a new *T. vaginalis* G3 assembly featuring six chromosome-length scaffolds derived from long-read sequencing. The new genome sequence data and corresponding assembly and annotation are all available at the NCBI [NCBI: Name, NYU_TvagG3_2, BioProject PRJNA16084, NCBI RefSeq assembly GCF_026262505.1; also TrichDB (Aurrecoechea et al., 2009): release 65, 14/09/2023]. These updated data are referred to here as the G3_2022 data. Supplementary Table 1 provides a lookup table between the G3_2007 and G3_2022 annotations providing continuity with the previous assembly by including where possible the TrichDB IDs (i.e., TVAG_XXXXXX, TVAG_RG_XXXXX).

### 2.3 CAZy annotation

The general protein functional annotation from *T. tenax* (this study) and *T. vaginalis* (Carlton et al., 2007) genomes were enriched by submitting the predicted protein sequences to the CAZy annotation pipeline. The reported data in this study have a specific focus on glycoside hydrolases (GH), key Carbohydrate Active enZymes (CAZymes), and the carbohydrate-binding modules (CBMs), as these are more likely to represent potential virulence factors. Briefly, automated crunching was performed followed by manual curation of borderline cases and fragments as described for hundreds of annotated genomes and used for updating the CAZy database (Lombard et al., 2014). Predictions of enzyme activities for *Trichomonas* spp. GHs, based on significant similarities with entries from a library of experimentally determined enzymatic activities of CAZymes (Drula et al., 2022), are also reported (Drula et al., 2022).

### 2.4 Estimates of completeness

Genome annotation quality and the completeness of gene annotation were estimated and quantified using the Benchmarking for Universal Single-Copy Orthologs (BUSCO) tool (Manni et al., 2021). The annotated proteins of *T. tenax* were searched against selected near-universal single-copy orthologs from the eukaryotic database (eukaryota_odb10 v5.2.2) of the hierarchical catalog of orthologs (OrthoDB v10) (Kriventseva et al., 2007). BUSCO searches the query proteome for “complete – single copy,” “complete – duplicated,” “fragmented,” and “missing” orthologs within the proteome that are expected to be highly conserved among related species. The output generated from this evaluation is presented as the percentage of the total 255 BUSCOs in the eukaryote_odb10 lineage from the OrthoDB database.

### 2.5 Gene family analyses

We employed OrthoVenn3 (using the Orthofinder algorithm) (Emms and Kelly, 2015; Sun et al., 2023) to identify and compare orthologs in the protein sequence sets from *T. tenax* (integrated annotated proteins) and *T. vaginalis*. Orthofinder utilizes DIAMOND for identifying sequence similarity and DendroBLAST (Kelly and Maini, 2013) for gene tree inference.

We also performed a gene network analysis using EGN (Halary et al., 2013) to predict homologous gene families using the full list of annotated proteins from the genomes of *T. vaginalis* G3_2022 (Genbank accession GCA_026262505.1) and *T. tenax* (this study). Results of an all vs all BLASTP searches (E-value ≤ 1 × 10^−10^, percent identity >25%, aligned length of query and subject ≥90%) were clustered into networks with edges linking gene nodes according to BLAST hits. Thresholds for recording significant BLAST hits were an E-value of less than 1 × 10^−10^, percentage identity of greater than 25% and an alignment length of at least 90% of the length of both sequences. A selection of gene networks for complex gene families of interest was visualized. Figures were generated using Cytoscape using the yfiles organic layout algorithm (Shannon et al., 2003).

## 3 Results

### 3.1 Gene predictions, functional annotation and completeness of *T. tenax* annotations

An overview of the annotation of the two *T. tenax* Hs-4:NIH draft genome sequences and their level of completeness is provided in Table 1. The Supplementary Data 1, 2 are the GFF3 files of the annotation of the *T. tenax* 2019 and 2022 draft genomes, respectively. Integrating the two annotations predicted a total of 20,286 distinct protein-coding genes for the *T. tenax* strain Hs-4:NIH. A FASTA file with the 20,286 protein sequences is available as the Supplementary Data 3. The BUSCO completeness score of 50.6% for *T. tenax* integrated annotation (20,287 proteins) is similar to the *T. vaginalis* (2022 annotation), with a completeness score of 53%. Here we describe the functional annotation of a selection of *T. tenax* proteins with a focus on CAZymes and a selection of some of the best characterized candidate virulence factors by taking advantage of the more extensive annotation of *T. vaginalis* proteins (Carlton et al., 2007, 2010; Hirt et al., 2011; Riestra et al., 2022). Supplementary Table 2 lists the 20,286 *T. tenax* (strain Hs-4:NIH) proteins with their functional annotations and inferred protein sequences, derived from the integrated annotation of the 2019 and 2022 draft genome sequence data.

**Table 1:** Overview of selected characteristics and annotations for the two published *Trichomonas tenax* draft genome sequences.

Draft genome^*^	***T. tenax* (2019)**	***T. tenax* (2022)**
Isolate/Strain	Hs-4:NIH	
Assembly size (Mb)	46.74	63.38
No. of scaffolds	4,161	4,904
N50 (bp)	13,554	38,386
L50 (bp)	1,070	469
% GC (Genome)	34.6	33.70
Genome assemblies ID	PRJEB22701	ASM2309173v1
No. of protein coding genes	16,786	18,852
BUSCO score (complete)^**^	129	
BUSCO score (Missing or Fragmented)^**^	126	
BUSCO score (Complete %)^**^	50.6	
tRNAs	226	298
rRNAs	2	4

^*^Benabdelkader et al. (2019) and Yang et al. (2022).

^**^BUSCO score based on the integrated annotations (20,287 proteins) from the 2019 and 2022 draft genomes.

### 3.2 CAZy annotation for *T. tenax* and *T. vaginalis*

A specific CAZy annotation was performed for both *T. tenax* (annotation from this study) and *T. vaginalis* (G3_2007 annotation) (Supplementary Table 3). Only 21 *T. vaginalis* CAZy annotated entries (7%), for a total of 287 entries, were deprecated, or not yet vetted, in the most recent assembly and corresponding annotation, G3_2022 annotation (Supplementary Tables 1, 3). The majority of *Trichomonas* spp. CAZy entries correspond to CAZymes members of the GH class with 126 TtGHs and 181 TvGHs. There are 86 *T. tenax* glycosyltransferases (TtGT) and 91 *T. vaginalis* glycosyltransferases (TvGT) (Supplementary Table 3). There are far fewer polysaccharide lyases (PLs) with three TtPL and 14 TvPL (Supplementary Table 3). Three *T. tenax* proteins are predicted to contain a CBM domain only, without an identified CAZyme domain (two entries with one CBM20, and one entry with one CBM32). Similarly, there are only three proteins with a single CBM13 domain without an identified CAZyme domain in *T. vaginalis* (Supplementary Table 3). Of the 189 GH families currently recognized in CAZy (http://www.cazy.org/ April, 25th 2024) (named GH1 to GH189), a total of 26 families were identified among either of the two species (Table 2, Supplementary Table 3). Some of the *Trichomonas* spp. GH proteins have evidence of a signal peptide (SP) and/or a transmembrane domain(s) (TMD), suggesting that these CAZymes could act on glycans with extracellular locations or within an organelle (Table 2). Among the GH13 proteins (14 in *T. tenax* and 24 in *T. vaginalis*), two in each species contain a GH133 domain (Table 2, Supplementary Table 3). Seven families with 10 or more, entries in at least one *Trichomonas* species include the GH13, GH16, GH30, GH31, GH47, GH77 and GH163 (Table 2). Table 3 lists selected features for the GH families with the largest number of entries or that include one or more activities that comprise potential mucin-targeting enzymes (mucinases) (Labourel et al., 2023) or other host glycoproteins encoded by both *Trichomonas* species. Two families, GH5 and GH32, are specific for *T. vaginalis* with one entry for each family, and these are confirmed in the G3_2022 annotation (Table 2, Supplementary Table 3). Table 4 lists selected characteristics for these two *T. vaginalis* specific GHs families.

**Table 2:** Annotated candidate glycoside hydrolases (GH) for *T. tenax and T. vaginalis*.

GH family #^*^	***Trichomonas tenax*** **(Hs-4:NIH)**				***Trichomonas vaginalis*** **(G3_2007)**			
	**Number annotated**	**SP**	**TMD**	**SP** + **TMD**	**Number annotated**	**SP**	**TMD**	**SP** + **TMD**
GH1	2	1	1	0	2	0	1	0
GH2	6	2	0	0	5	2	0	0
GH3	7	2	1	0	4	0	1	0
GH5^a^	0	0	0	0	1	0	0	0
GH13	14	4	4	0	23	5	4	0
GH14	4	3	0	0	4	2	0	0
GH15	1	1	0	0	3	1	0	0
GH16	2	1	0	1	10	0	0	1
GH18	1	0	0	0	1	0	0	0
GH19	6	2	1	0	8	0	0	0
GH20	6	2	2	1	6	0	2	0
GH25	5	0	0	0	4	0	0	0
GH27	2	1	1	0	1	0	0	0
GH29	2	1	0	1	2	0	2	0
GH30	1	1	0	0	16	0	0	0
GH31	17	6	2	3	17	3	2	1
GH32^a^	0	0	0	0	1	0	0	0
GH33	1	1	0	0	2	0	0	0
GH36	2	1	1	0	2	0	1	0
GH37	1	0	0	0	2	0	0	0
GH38	4	0	2	0	5	0	0	0
GH47	24	11	8	1	27	1	14	0
GH77	7	0	1	0	14	0	0	0
GH99	8	3	1	0	6	1	2	0
GH133 (GH13)	2	0	0	0	2	0	0	0
GH163	2	0	1	1	10	1	4	3
Total GH domains^**^	127	43	26	8	178	16	33	5

^*^SP, signal peptide; TMD, transmembrane domain.

^a^GH families restricted to *T. vaginalis*.

^**^Number of GH families corresponding to 125 and 176 distinct proteins for *T. tenax* and *T. vaginalis*, respectively, with two proteins with the configuration GH13-GH133 in each species.

**Table 3:** Selected characteristics of glycoside hydrolase families with 10 or more entries or known to include enzymes targeting mucins or other host glycoproteins, encoded by both *T. tenax and T. vaginalis*.

GH family #^*^	**Known enzymatic activities(s)**	**Comments**	**References**
GH2 (6/5)	16 different known activities, including β-galactosidases and β -mannosidase, the two predicted activities for the enzymes of the two species	Mostly bacterial and a few fungal enzymes. Include mucinases.	Lombard et al., 2014; Labourel et al., 2023
GH16 (2/10)	21 different activities, including endo-β-1,4-galactosidases and endo-β-1,3-glucosidases	Mostly bacterial and some fungal enzymes. Classified in 23 subfamilies. Include mucinases.	Viborg et al., 2019; Labourel et al., 2023
GH29 (2/2)	Eight different known activities targeting fucose containing substrates, including α-1,4-L-fucosidase	Mostly bacterial and some few fungal enzymes. Include mucinases.	Lombard et al., 2014; Labourel et al., 2023
GH30 (1/16)	14 different known activities, including β-1,6-glucosidase and Endo-β-1,4-xylanase	Mostly bacterial and some fungal enzymes. Classified in 10 subfamilies	Lombard et al., 2014
GH31 (17/17)	17 different known activities including α-galactosidase and α-mannosidase	Mostly bacterial and a few fungal enzymes. Classified in 20 subfamilies. Include mucinases.	Lombard et al., 2014; Arumapperuma et al., 2023; Labourel et al., 2023
GH33 (1/2)	Five different known activities including sialidases	Mostly bacterial and a few fungal enzymes. Include mucinases.	Lombard et al., 2014; Labourel et al., 2023
GH47 (24/27)	Mannosyl-oligosaccharide α-1,2-mannosidase	Mostly eukaryotic and a few bacterial enzymes	Lombard et al., 2014
GH77 (7/14)	4-α-glucanotransferase/amylomaltase	Mostly bacterial and a few eukaryotic and archaeal enzymes	Lombard et al., 2014
GH163 (2/10)	Endo-like activity targeting the β-GlcNAc–mannose from complex N-glycans	Mostly bacterial enzymes. Could target mammalian glycoproteins, including immunoglobulins such as secretory IgA	Briliute et al., 2019

^*^Values in brackets (x/y) are the number of annotated genes encoding listed GH families for respectively *T. tenax* and *T. vaginalis*.

**Table 4:** Selected characteristics of the two GH families restricted to *T. vaginalis*.

GH family #^*^	**Known enzymatic activities(s)**	**Comments**	**References**
GH5_4 (1 entry)	30 different known activities. GH5 subfamily 4: endo-β-1,4-glucanases, licheninases, and xylanases	Classified in 57 subfamilies GH5 subfamily 4: typically, extracellular bacterial enzymes, also found among some ciliates and fungi.	Aspeborg et al., 2012
GH32 (1 entry)	15 different known activities on a broad range of fructose-containing substrates	Mostly bacterial and a few fungal enzymes	Lombard et al., 2014

### 3.3 Identification of candidate virulence factors in *T. tenax* through comparisons with *T. vaginalis*

#### 3.3.1 Peptidases

Similar to the 469 predicted peptidases in *T. vaginalis* (Carlton et al., 2007), a large number of candidate peptidases (476) were identified in *T. tenax* (Supplementary Table 4). These include 240 cysteine peptidases, 141 metallopeptidases, 85 serine peptidases and seven aspartic peptidases (Supplementary Table 4). A selection of *T. tenax* peptidase families, including some of those with the largest number of homologs to *T. vaginalis* factors and that are experimentally demonstrated to contribute to host damage or are candidate virulence factors, are listed in Table 5. A number of these peptidases represent candidate secreted or cell surface proteins based on the presence of inferred signal peptides and/or TMD, consistent with a potential role in targeting extracellular or phagocytosed proteins from host or microbiota cells (including peptidoglycans) (Supplementary Table 4, Table 5). The *T. tenax* genome encodes a large number (100 members) of family C19 ubiquitin hydrolases, similar to *T. vaginalis* (117 members) (Carlton et al., 2007) (Supplementary Table 4). In *T. vaginalis* > 50% of the C19 ubiquitin hydrolases are likely to be functional (Hirt et al., 2011). This large number of C19 peptidases that are known to be functionally associated with the 20S proteasome (ubiquitin C-terminal hydrolases) in model systems (Barrett and Rawlings, 2001) highlight for these two species the importance of cytosolic protein degradation.

**Table 5:** Selected annotated peptidase families representing candidate virulence factors of *T. tenax*.

**Clan**	**Family**	**Number**	**Example from family**	Candidate virulence factors^*^
CA	C1	46	Papain	Figueroa-Angulo et al., 2012
CA	C40	7	NlpC/P60	Barnett et al., 2023
MA	M8	36	Leishmanolysin/GP63	Ma et al., 2011; Figueroa-Angulo et al., 2012
MA	M60	13	Enhancin	Hirt et al., 2011; Nakjang et al., 2012; Riestra et al., 2022
SB	S8	31	Subtilisin	Hernández-Romano et al., 2010

^*^Listed references are either reviews or original publications describing the listed peptidases as virulence factors. These were either directly demonstrated to degrade host proteins or damage host cells (C1 peptidases) or through indirect mechanisms by targeting members of the microbiota (C40 peptidases). M60-like peptidases are speculated to represent virulence factors based on their cell surface location in *T. vaginalis* and enriched distribution among mammalian host associated microbial pathogens and members of the microbiota. S8 peptidases are speculated to represent virulence factors based on their cell surface location in *T. vaginalis* and known targets among several microbial pathogens including parasites, fungi and bacteria.

#### 3.3.2 Surface proteins as adhesins

**Figure 1:**
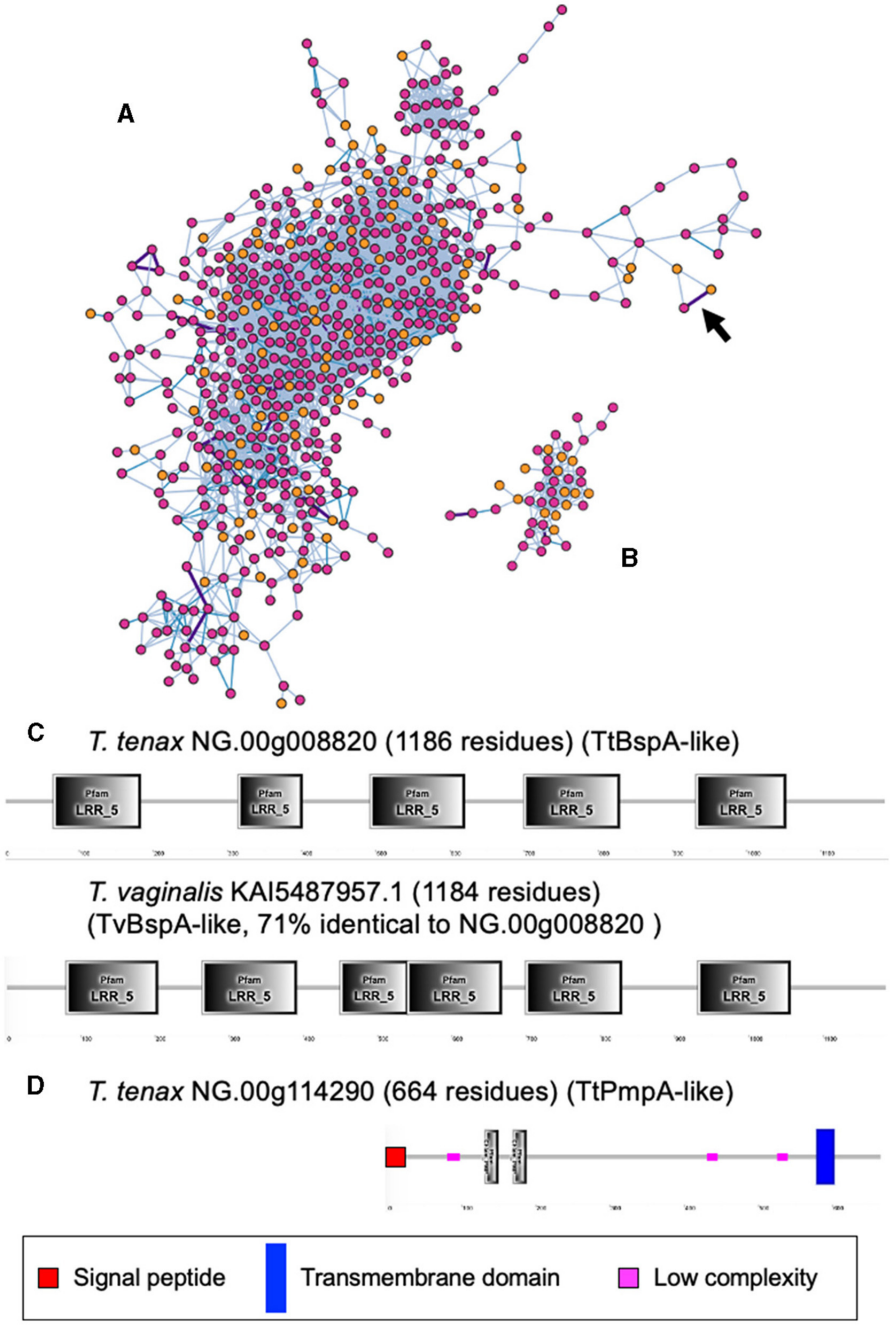
Sequence comparisons between *T. tenax* and *T. vaginalis* BspA- and Pmp-like proteins. The gene networks illustrate the relationships between **(A)** BspA- and **(B)** Pmp-like protein families. Gene networks in which nodes represent individual homologous genes and edges represent significant BLASTP alignments. Nodes are colored according to species (pink: *T. vaginalis*, orange: *T. tenax*). Edge color is scaled according to BLASTP alignment percentage identity from 25–40% (light blue), 40–70% (blue) and >70% (purple). Purple edges are displayed with greater width to aid visibility. The two illustrated networks correspond to the one with the largest number of nodes (genes) for the respective gene families. The arrow in **(A)** indicates the edge between the two BspA-like sequences with the highest level of sequence similarity between *T. tenax* and *T. vaginalis* proteins **(C)**. **(C)** The structural organization inferred by SMART for the two most similar BspA-like proteins (71% identity) between *T. tenax* and *T. vaginalis* are illustrated and drawn to scale. Not all domains identified by SMART are illustrated due to overlap between some of these inferred domains. None of these proteins have an inferred SP or TMD. **(D)** The structural organization inferred by SMART for one *T. tenax* Pmp-like protein, which is inferred to possess both a SP and a TMD, with the segment with the Pmp domain (motif GGA[ILV] and FXXN) to be inferred to be exposed to the extracellular environment.

**Figure 2:**
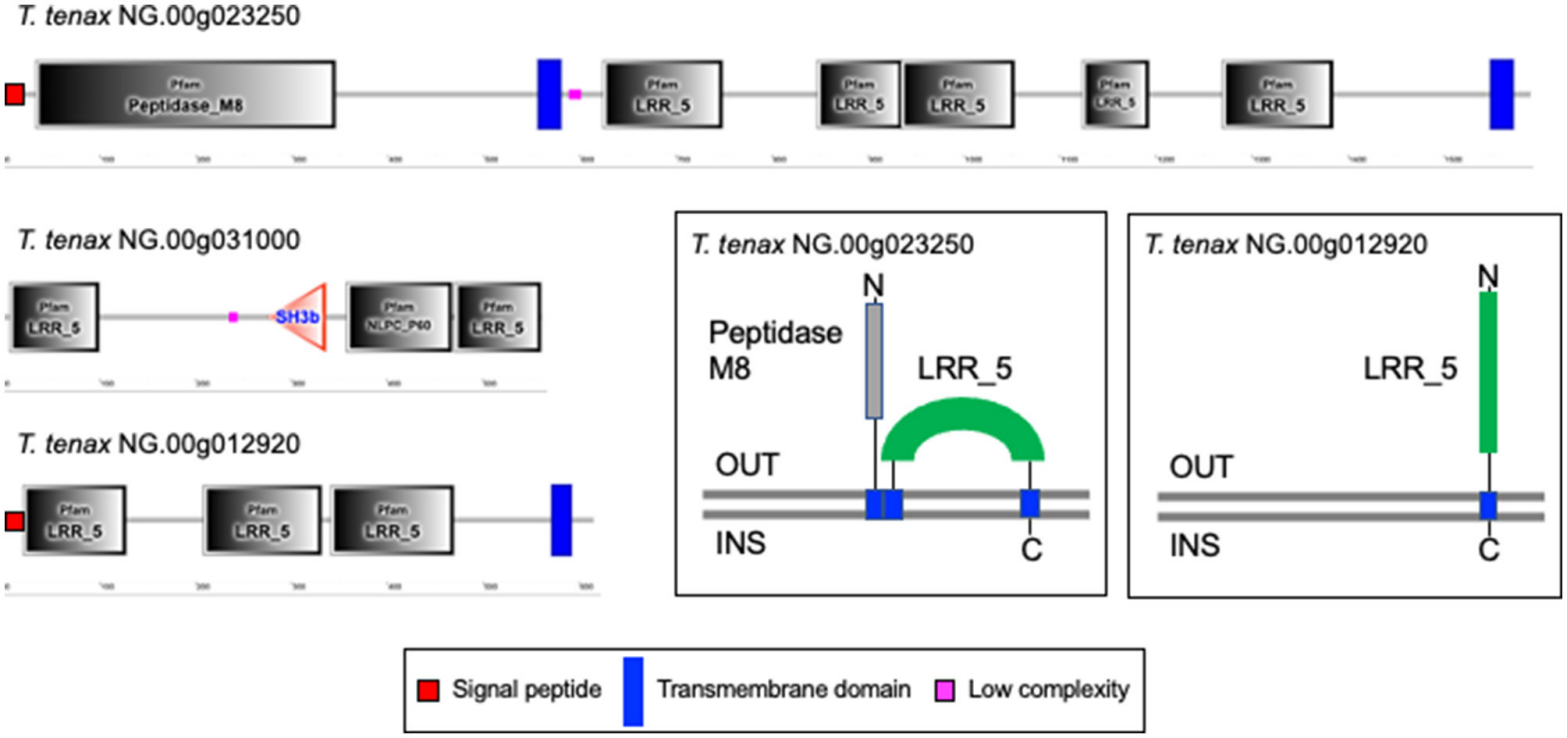
Examples of TtBspA-like proteins. The graphs represent SMART outputs drawn to scale. Two illustrated entries were selected as they possess a peptidase domain: respectively a metallopeptidase M8, leshmaniolysin domain, and a cysteine peptidase C40, NlpC/P60 domain. The entry with the metallopeptidase M8 domain has two (TMHMM—SMART output, illustrated) or three (DeepTMHMM output, see box) inferred TMDs. The topological configuration inferred from DeepTMHMM has the peptidaseM8 domain and the LRR domain to be extracellular, as illustrated within the box. These are not drawn to scale and only illustrate protein topology in relation to the bilayer of the plasma membrane of *T. tenax*. DeepTMHMM also inferred a SP, which was not identified by SMART. The C40 NlpC/P60 peptidase possess the key catalytic triad (C-H-H) and one inferred SH3b domain, as identified for other *Trichomonas* NlpC/P60 (Barnett et al., 2023). The protein *T. te*nax NG.00g012920 has the more common and characteristic structural organization and topology of BspA-like proteins among *Trichomonas* and relatives (Noël et al., 2010; Handrich et al., 2019) and possess both a SP and a TMD. The LRR domain is inferred to be exposed to the extracellular environment, consistent with a potential binding role of the LRR domain to one or more extracellular ligands, such as molecules on the host epithelial or bacteria cell surface or a specific soluble molecule. OUT, outside the cell; INS, inside the cell. The symbols for SP, TMD and low complexity sequences are indicated.

**Figure 3:**
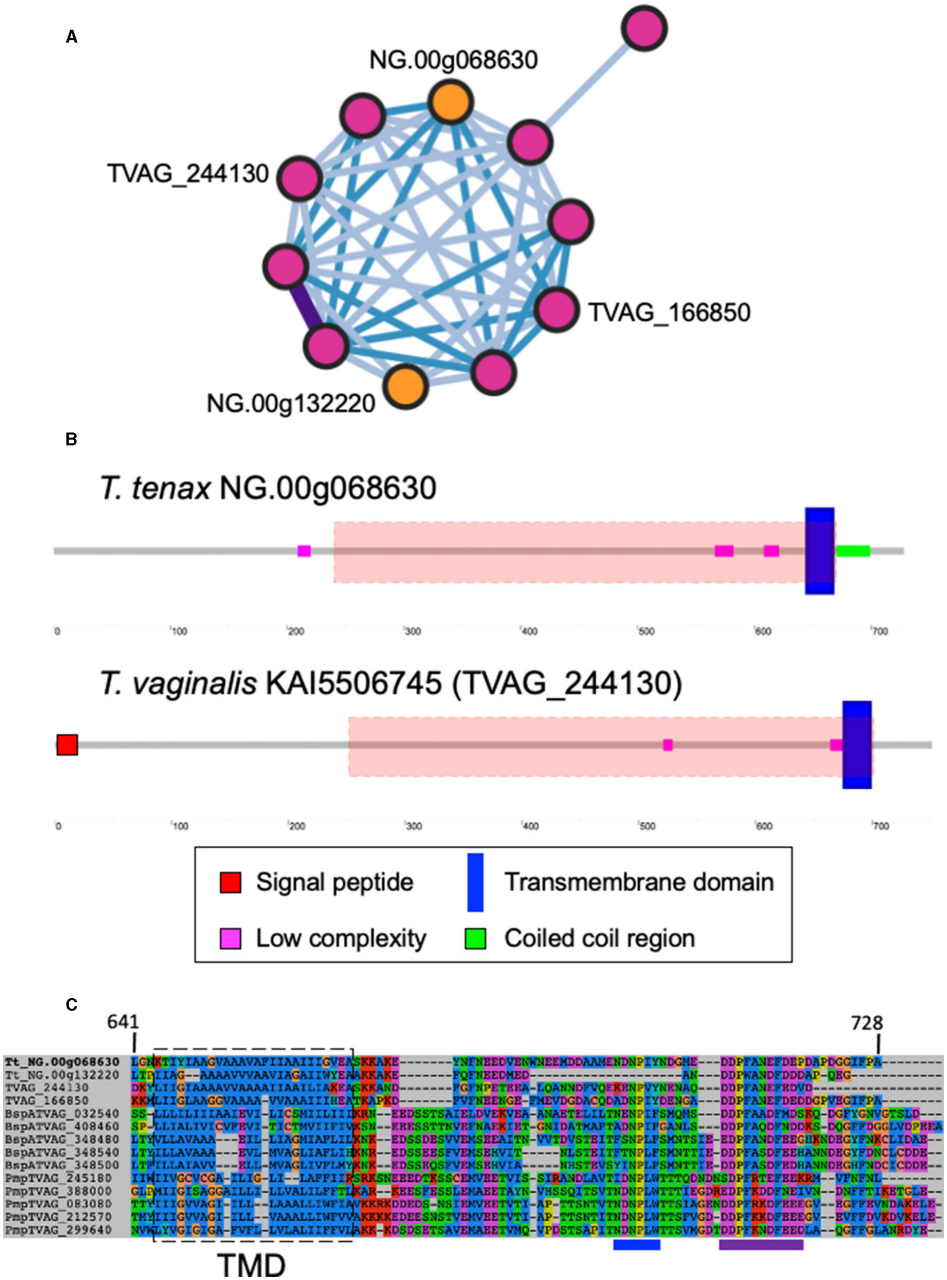
Sequence comparisons between *T. tenax* and *T. vaginalis* BAP-like proteins. **(A)** Gene network in which nodes represent individual homologous genes and edges represent significant BLASTP alignments. Nodes are colored according to species (pink: *T. vaginalis*, orange: *T. tenax*). Edge color is scaled according to BLASTP alignment percentage identity from 25-40% (light blue), 40-70% (blue) and >70% (purple). Purple edges are displayed with greater width to aid visibility. The locus tags for the two TtBAP-like annotated proteins member of the illustrated network are indicated as are the two *T. vaginalis* BAP-like proteins (2007 locus tag) with functional data (see main text). **(B)** The structural organization inferred by SMART for one *T. tenax* and one *T. vaginalis* BAP-like are illustrated and drawn to scale. The symbols for SP, TMD, low complexity and coiled coil sequences are indicated. The red dashed boxes illustrate the position of the HHPred top hit alignments between the alignment of 13 selected BAP-like proteins used as query: “Cell surface glycoprotein; S-layer csg, STRUCTURAL PROTEIN; HET: CA, BGC; 3.46A {Haloferax volcanii DS2}”, probability: 98.34, e-values: 0.0053. The alignment starts and ends at position 222 and 668, respectively, from NG.00g06863. **(C)** Multi sequence alignment illustrating the conservation of the TMD and cytoplasmic tail for selected BAP-like, BspA and Pmp-like proteins. The approximate position of the inferred TMD is indicated by the dashed box and the position of the [FY]NPX[YF]-like motif and the acidic cluster are indicated by respectively a blue and purple line below the alignment, these could represent signal for endocytosis (see Hirt et al., 2011; Handrich et al., 2019).

A large number of genes were annotated *in T. vaginalis* to encode candidate surface proteins mediating key interactions with its environment, including host epithelial cells (adhesins) and members of the microbiota (Carlton et al., 2007; Hirt et al., 2011; Riestra et al., 2022). Several of these proteins have been experimentally demonstrated in *T. vaginalis* to be expressed on the cell surface and to mediate binding to host cells (Hirt et al., 2011; Riestra et al., 2022). We focus here on some specific examples which indicate that *T. tenax* has a similar repertoire of candidate binding factors to host cells, and potentially to members of the oral microbiota. These include 244 TtBspA-like (Supplementary Table 5) and 24 TtPmp-like (Supplementary Table 6) proteins (Figure 1). Two TtBspA-like proteins contain a peptidase domain, including an M8 peptidase and a NlpC/P60 peptidase (Figure 2). There is also evidence for at least 14 *T. tenax* BAP-like proteins related to TVAG_244130 (de Miguel et al., 2010), with extracellular domains that are shared with bacterial proteins (Table 6). Two of the TtBAP-like proteins have similar structural organization with eight TvBAP-like proteins (>25% identify level, similar protein length and shared TMD-CT), including the TvBAP-like proteins TVAG_166850 and TVAG_244130 (Figure 3).

**Table 6:** *T. tenax* encodes BAP-like homologs.

**Taxa**	**Habitat and relationship with host**	**Top bit score (locus tag)**	**Top e-value**	**Top hit protein length**	**Total number of hits**
*Trichomonas vaginalis* G3	Urogenital tract-pathobiont (human)	348 (TVAG_335250)	6e^−105^	751	60
*Trichomonas tenax* Hs-4:NIH	Oral cavity- pathobiont? (human, dog, cat)	303 (NG.00g068630)	3e^−92^	728	14
*Clostridium* sp. CAG:273	Gut-normal flora (human)	102 (BN581_00590)	7e^−18^	1401	1
Clostridia bacterium	Gut-normal flora (human)	99.8 (UIT70_01140)	3e^−17^	652	1
*Eubacterium* sp.	Gut-normal flora (mouse)	100 (K2K71_04565)	4e^−17^	1027	1
*Clostridium* sp. CAG:245	Gut-normal flora (human)	98.6 (BN559_00332)	1e^−16^	893	1
*Clostridium* sp. CAG:245_30_32	Gut-normal flora (human)	95.1 (BHW09_08500)	1e^−15^	893	1

The shown bit scores, e-values and number of hits are derived from BLASTP searchers (e-value ≤ e^−10^) at the NCBI Blast server using the *T. vaginalis* BAP-like protein TVAG_244130 sequence as query against the (i) nr database or (ii) the 20,286 annotated proteins from *T. tenax* Hs-4:NIH. Only the features of the top hits are listed for each taxon. The ranking, from top to bottom of the table, is according to the bit score, with the highest value on the top.

#### 3.3.3 Saposin-like proteins

**Figure 4:**
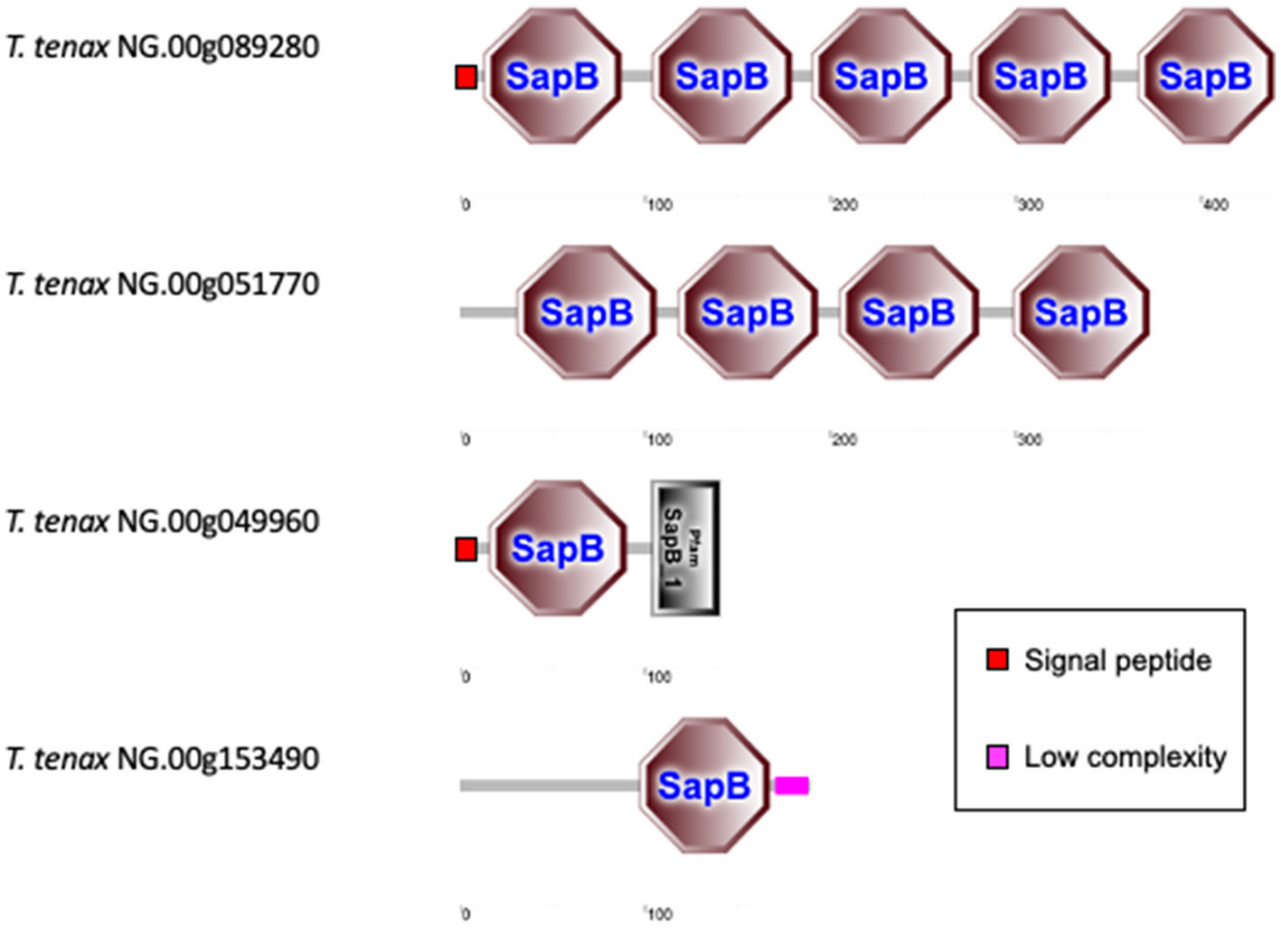
Examples of TtSAPLIP-like protein structural diversity. The graphs represent SMART outputs drawn to scale. The four illustrated examples focus on the proteins with multiple SAPLIP domains (five and four) and two examples of proteins with two or one inferred SAPLIP domains only. The remaining six TtSAPLIP-like proteins have only one inferred SAPLIP. The symbols for SP and low complexity sequences are indicated.

The *T. vaginalis* G3_2007 annotation identified 12 candidate pore-forming effectors that belong to the conserved family of saposin-like proteins (SAPLIP) (named TvSAPLIP1-12) that represent important candidate virulence factors (Carlton et al., 2007; Hirt et al., 2011; Diaz et al., 2020). We identified 10 SAPLIP *T. tenax* encoding genes (Supplementary Table 7). Three TtSAPLIP proteins display multiple domains with five, four and two different SAPLIP domains (Figure 4). The presence of a SP was inferred for seven TtSAPLIP, consistent with extracellular functions (Supplementary Table 7, Figure 4).

#### 3.3.4 Extracellular vesicles

As carefully reviewed by Riestra et al. (2022), there are published evidence, including electron microscopy, proteomics surveys and cell biology, demonstrating that *T. vaginalis* can secrete two types of extracellular vesicles (EVs), exosomes and microvesicles (MVs) and that these modulate several important aspects of *T. vaginalis*-host cells interactions (Twu et al., 2013; Rai and Johnson, 2019; Artuyants et al., 2020; Molgora et al., 2023; Salas et al., 2023). We identified *T. tenax* homologs for the Endosomal Sorting Complex Required for Transport (ESCRT) machinery made of four complexes (complexes I to IV) (Leung et al., 2008), which are involved in exosome formation in multi-vesicle bodies (MVBs) and secretion in many eukaryotes, strongly suggesting that *T. tenax* also possess MVBs and could secrete exosomes (Supplementary Table 8).

## 4 Discussion

### 4.1 CAZymes

CAZymes, including GHs, are of great interest as their glycans substrates are widely distributed across all cellular life forms as well as viruses. Glycans mediate a myriad of biological functions, including handling of carbon reserves, structural roles, or as mediators of intra- and intercellular interactions between organisms or organisms and viruses. CAZymes and GH in particular, but also CBM-containing proteins, include virulence factors of numerous cellular and viral pathogens (Garron and Henrissat, 2019; Wardman et al., 2022). A diverse array of GH families was identified in the two *Trichomonas* species and the majority of these are shared between them, suggesting that these play key roles in the biology of both the oral cavity and the urogenital tract for *T. tenax* and *T. vaginalis*, respectively. A number of families are characterized by relatively larger membership with more than 10 members in one or both species. Three families, GH13, GH31 and GH47 stand out as making up the largest families present in both species (ranging from 14 to 27 entries), further emphasizing their potential importance in the mucosal lifestyle of the respective species.

Notably, some GH entries represent candidate virulence factors. Seven GH families (GH2, GH16, GH18, GH16, GH29, GH33 and GH163) could include members from either species that target mucins (Labourel et al., 2023) (candidate endo-β-N-acetylglucosaminidases in GH163, candidate fucosidases in GH29, candidate sialidases in GH33, candidate β-galactosidases in GH2), important structural proteins at mucosal surfaces mediating key innate immune functions in host-microbe interactions (Hansson, 2020). *T. vaginalis* might be able to degrade mucins more extensively as it has candidate O-glycan endo-β-1,4-galactosidases in GH16, while *T. tenax* does not seem to encode a homolog with the same predicted activity. Mucins are typically targeted by mucosal pathogens as well as some members of the microbiota (Labourel et al., 2023). One or more of these enzymes could also target SIgA, a key immunoglobulin in the oral cavity (Feller et al., 2013) and the urogenital tract (Garcia et al., 2015), an activity demonstrated for a GH163 from a common member of the human gut microbiota (Briliute et al., 2019).

Several GH families could also target members of the microbiota cell walls, either of bacteria (lysozymes GH19 and GH25) or fungi (chitinase GH19) or GH47, which target glycoproteins containing α-1,2-linked mannose residues. Candidate lysozymes could be functional partners of the NlpC/P60 peptidases, which were demonstrated to target peptidoglycans (Barnett et al., 2023), to contribute to deconstruct bacterial cell walls. These sets of enzymes could be important for the two *Trichomonas* species to target bacteria for interspecies competition and extraction of important source of nutrients. As fragments of peptidoglycans represent strong pro-inflammatory molecules (Irazoki et al., 2019), the products of the combined activities of these enzymes (GH and peptidases) could also be an important factor contributing to inflammation, such as the damaging inflammations characteristic of both periodontitis (Baker et al., 2024) and trichomoniasis (Mercer and Johnson, 2018).

As most of these GH families are known to mediate multiple distinct enzymatic activities, it will be important to study their exact substrate specificities, expression profile and cell biology to experimentally establish their functional importance for *Trichomonas* species.

### 4.2 Candidate surface proteins and exosomes

Several key aspects of host-microbe interactions are mediated by cell surface proteins and EVs, such as exosomes, which modulate cell-cell interactions and contact-dependent cytolysis (e.g., Hirt et al., 2011; Riestra et al., 2022). As for *T. vaginalis* (Carlton et al., 2007; Hirt et al., 2011; Handrich et al., 2019; Riestra et al., 2022), the annotation of *T. tenax* proteins identified a large number of candidate surface proteins including members of TtBspA-like, TtPmp-like and TtBAP-like families. Two TtBspA-like proteins possess a peptidase domain (one with an M8 metallopeptidase domain and the other with NlpC/P60 cysteine peptidase domain), a configuration distinct from the single TvBspA-like protein with a peptidase domain (a partial serine peptidase S8/S53-like domain) (Noël et al., 2010). Potential surface proteins also include several candidate peptidases and GHs, as they possess inferred TMDs. There is transcriptional evidence for the expression of 81 TtBspA-like and 11 TtPmp-like encoding genes (Handrich et al., 2019). In *T. vaginalis*, one or more members of these three families have been experimentally demonstrated to be expressed on the cell surface and to mediate interactions with vaginal epithelial cells (de Miguel et al., 2010; Handrich et al., 2019). Hence some of the identified homologs from these protein families in *T. tenax* could contribute to interactions of this species with epithelial cells of the oral cavity, interactions which are likely key to mediating the long-term colonization of the periodontal pocket, and that could also contribute to cell contact-mediated cytolysis of host cells (Ribeiro et al., 2015; Matthew et al., 2023). Our gene network analyses that used stringent coverage of the pairwise alignment length criteria (≥90% coverage) indicate that a number of BspA-, Pmp and BAP-like entries have similar structural organization, with a number of these sharing similar length and a TMD and cytoplasmic tail (TMD-CT). Visual inspection of the cytoplasmic tail of TtBAP- and TvBAP-like proteins identified conserved TMD-CT shared with some TvBspA- and TvPmp-like proteins (Hirt et al., 2011; Handrich et al., 2019). This indicates that the gene fusion events underlying these shared TMD-CT domains between BspA and Pmp-like coding genes (Hirt et al., 2011) have also involved some members of the BAP-like family (Handrich et al., 2019) and that these fusion events might have occured in a common ancestor to *T. tenax* and *T. vaginalis*. This further supports the functional importance of this type of TMD-CT and in the context of various unrelated extracellular domains: (i) BspA-like proteins characterized by a specific type of leucine-rich repeat (TpLRR), (ii) Pmp-like repeats (that include two specific motifs GGA[ILV] and FXXN) and (iii) BAP domain (related to “Cell surface glycoprotein; S-layer”). The two TvBAP-like proteins (TVAG_166850, TVAG_244130) were shown to be significantly more highly expressed by more adherent *T. vaginalis* strains compared to less adherent strains (de Miguel et al., 2010). Overexpression of some members of these three gene families, one TvBspA, two TvPmp (Handrich et al., 2019) and two BAP-like proteins (TVAG_166850, TVAG_244130) (de Miguel et al., 2010) significantly increased *T. vaginalis* binding to vaginal epithelial cells in *in vitro* binding assays, suggesting that some members of the TtBspA-like, TtPmp-like and TtBAP-like proteins families could contribute to *T. tenax* binding to oral epithelial cells. The Tv/TtBspA-like proteins could also mediate interactions between the *Trichomonas* species and members of the microbiota based on their known functions in bacteria-bacteria interactions among oral bacterial pathogens (Sharma, 2010), which could contribute to bacteria phagocytosis, as previously speculated for *T. vaginalis* (Noël et al., 2010). Notably, the TtBspA-like proteins could also contribute to the inflammatory response of the periodontal pocket, as demonstrated for some oral bacterial BspA proteins (Sharma, 2010).

Some members of these protein families also contribute to the proteome of *T. vaginalis* exosomes (Riestra et al., 2022; Ong et al., 2024). Our identification of homologs of subunits of the ESCRT machinery (with homologs identified for all four complexes I to IV), suggests that exosomes are likely produced and secreted by *T. tenax*. Hence it will be important to affirm the presence and functional characteristic of EVs in *T. tenax*, including their protein and RNA complement as has been done for *T. vaginalis* (Riestra et al., 2022; Matthew et al., 2023; Ong et al., 2024). These potentially include members of the BspA-like proteins and peptidases (e.g. some calpain-like cysteine peptidase and metallopeptidases, see next section). These different proteins possibly play multiple roles, including modulating *T. tenax* binding properties to host cells, and mediating cytolytic capabilities, which could both contribute to the pathobiology of periodontitis. Other proteins identified in *T. vaginalis* exosomes include membrane protein tetraspanins and CBM20-GH77 enzymes (candidate 4-α-glucanotransferase) (Rai and Johnson, 2019). We identified homologs of these proteins in *T. tenax*, including 15 tetraspanins of which 10 were inferred to possess the canonical four TMDs of tetraspanins (Supplementary Table 2) and five proteins with the CBM20-GH77 domain configuration, with one protein inferred to possess a TMD (Supplementary Table 3). In *T. vaginalis* some tetraspanin proteins were also shown to mediate *T. vaginalis* binding to self (Cóceres et al., 2015), thus contributing to swarming, which is thought to have implication for its virulence (Hirt, 2013). Notably, the CBM20 domain from one of the *T. vaginalis* GH77 exosomal proteins was demonstrated to bind to heparan sulfate from host proteoglycans (Rai and Johnson, 2019). Hence these different proteins represent primary targets to study *T. tenax* interactions with oral epithelial cells and with itself. Cell surface proteins and EVs could also play important roles in *T. vaginalis* and *T. tenax* interactions with immunocytes and members of the microbiota or extracellular matrix (ECM) proteins.

### 4.3 Candidate peptidases and pore-forming proteins

Peptidases are considered to mediate and modulate important aspects of *T. vaginalis* cytoadherence and host-cell cytolysis, and to degrade specific host proteins including immunoglobulins, the complement, ECM proteins and potentially some host-produced anti-microbial peptides (Carlton et al., 2007; Hirt et al., 2011; Figueroa-Angulo et al., 2012; Fichorova et al., 2013; Riestra et al., 2022).

Hence these enzymes are of primary interest to investigate the molecular basis of *T. tenax* pathobiology. Indeed, initial investigations indicated the secretion of cysteine peptidases and metallopeptidases by *T. tenax* (reviewed in Matthew et al., 2023). Like *T. vaginalis* (Carlton et al., 2007; Hirt et al., 2011), *T. tenax* is endowed with a large number and broad diversity of peptidases. A number of these could be either secreted as soluble proteins, secreted as exosomes cargo, or expressed on the cell surface or within organelles such as the lysosomes, where they could contribute to the degradation of proteins on the cell surface of the parasite or from phagocytosed microbial or human cells. Several M8 peptidases have an inferred SP and/or one or more TMD. One member of the M8 family was characterized with an LRR BspA-like containing domain and inferred to possess three TMDs and exposed to either the cell surface or lumen of an organelle, a location consistent with the function of the related M8 peptidases, including Leishmanolysin, an important virulence factor among *Leishmania* species (Hirt et al., 2011).

In addition to peptidases potentially targeting host and microbial proteins, some peptidases are also likely to specifically target peptides of bacterial peptidoglycans. These include the NlpC/P60 peptidases, and initial analyses of the first published draft genome of *T. tenax* (Benabdelkader et al., 2019) identified six genes encoding bacteria-like TtNlpC/P60 peptidases (Barnett et al., 2023). The genes encoding these NlpC/P60 peptidases were likely acquired by two distinct lateral gene transfers events from distinct gram-positive bacteria donors into a common ancestor of *Trichomonas* species as these are found in the genomes of *T. vaginalis, T. gallinae* (Alrefaei et al., 2019) and *T. tenax* (Barnett et al., 2023). A total of seven distinct TtNlpC/P60 encoding genes were identified when annotating the more recent draft genome (Yang et al., 2022). The latest identified TtNlpC/P60 protein is characterized by a distinct structural configuration, which include two segments of LRR BspA-like proteins, one on each end of the protein. As the LRR of some cell surface BspA-like proteins from oral bacteria mediate bacteria-bacteria interactions (Sharma, 2010), these two LRR segments could target this peptidase to the cell surface of specific bacteria. Six of the seven TtNlpC/P60 have inferred SP, consistent with digesting extracellular targets, such as the peptidoglycans from bacteria. The identification of several candidate GHs potentially targeting the glycans from peptidoglycans (e.g., TtGH25, candidate lysozymes) suggests that the TtNlpC/P60 protein could be functional partners of these CAZymes to effectively deconstruct peptidoglycans from the bacterial members of the oral and vaginal bacterial microbiota. As peptidoglycan fragments derived from the combined activity of peptidases and CAZymes are very strong pro-inflammatory molecules (Irazoki et al., 2019), these enzymes could represent important indirect virulent factors contributing to inducing damaging inflammations characteristic of periodontitis (Baker et al., 2024) and trichomoniasis (Mercer and Johnson, 2018; Riestra et al., 2022). In addition, as *T. vaginalis* and *T. tenax* are associated with dysbiotic microbiota, the potential preferential targeting of mutualistic bacteria by these enzymes, such as *Lactobacillus* species (that have anti-inflammatory properties and other important protective functions) typically affected/eradicated by *T. vaginalis* infections, these enzymes could also contribute to the worsening of the inflammatory response of the oral and vaginal mucosa.

The 10 TtSAPLIP candidates pore-forming proteins are also potentially important virulent factors for *T. tenax* as discussed for *T. vaginalis* (Hirt et al., 2011; Diaz et al., 2020). The functional characterization of the TvSAPLIP12 demonstrated that it is haemolytic and cytotoxic toward epithelial cells and the bacterium *Escherichia coli* (Diaz et al., 2020). The inference of SP for the majority of the TtSAPLIP proteins is consistent with the hypothesis that these candidate virulence factors are secreted and able to act as pore–forming proteins targeting host cells and/or members of the microbiota as shown (or suggested) for the TvSAPLIP proteins (Hirt et al., 2011; Diaz et al., 2020; Margarita et al., 2022).

## 5 Conclusions

Our detailed integrated annotations of the two *T. tenax* draft genomes from the currently single strain available from the American Type Culture Collections (ATCC, strain Hs-4:NIH) further support a model where *T. tenax* is a pathobiont that directly and/or indirectly contribute to periodontitis (Ribeiro et al., 2015; Marty et al., 2017; Benabdelkader et al., 2019; Bisson et al., 2019; Matthew et al., 2023). Comparison between *T. tenax* and *T. vaginalis* annotations also identified some novel shared candidate virulence factors including GHs potentially targeting host or bacterial glycans, making them relevant in both species, and warranting further study in both species. Based on the features of the protein complement of *T. tenax* shared with *T. vaginalis*, the oral protist could contribute to worsening the prognosis and/or initiating periodontitis in multiple ways including through host cell cytolysis and bacteria targeting, which together could contribute synergistically at inducing and/or amplifying damaging inflammations. Periodontitis is primarily recognized as a condition driven by bacterial dysbiosis, which is sufficient to develop the condition (Baker et al., 2024). However, the high prevalence of *T. tenax* among humans and pets (dogs and cats) and the heterogeneity of disease prognosis between individuals makes it potentially highly relevant to numerous cases of periodontitis and could help also at stratifying patients (Marty et al., 2017). Hence, affected hosts could potentially benefit from treatments targeting both *T. tenax* and pathogenic bacteria to reduce/eliminate the damaging inflammation characteristic of periodontitis. Establishing the potential causality of *T. tenax* for periodontitis will require further investigation, including longitudinal studies among humans and animals, and the molecular and cellular characterization of several candidate virulence factors. A notable virulence factor associated with *T. vaginalis* are the *Trichomonas vaginalis* viruses (TVV), which are members of the totoviridae known to have strong pro-inflammatory properties (Fichorova et al., 2013). Hence, it will be also very interesting to investigate the potential presence and relevance of related TVV-like viruses in *T. tenax* isolated from humans and pets. Furthermore, *T. vaginalis* is associated with two mycoplasma-like endosymbiotic bacteria, which are known to contribute to promoting *T. vaginalis* growth and to boosting virulence by up-regulating adhesion to human epithelial cells and haemolysis in *in vitro* conditions (Margarita et al., 2022). It is currently unknown if sets of related endosymbionts are associated with *T. tenax*. It will be in particular important to sequence genomes from across the spectrum of the known genetic diversity of *T. tenax*, which includes specific genotypes associated with worse periodontitis (Benabdelkader et al., 2019). This should include recent clinical isolates from both humans and animals (e.g. dogs and cats), as the existing strain (Hs-4:NIH) has been grown i*n vitro* since 1959, and thus may not represent the best, albeit a very valuable and important, model system to study *T. tenax*-host interactions.

## Data availability statement

The original draft genome sequence data analyzed in this study are available at the NCBI (NCBI Genome assemblies PRJEB22701 and ASM2309173v1). All the key outputs of the sequence analyses for this study can be found in the Supplementary material. They are all described in the main text and in the Supplementary material.

## Author contributions

LM: Conceptualization, Data curation, Formal analysis, Investigation, Validation, Visualization, Writing – original draft, Writing – review & editing. AH: Data curation, Formal analysis, Investigation, Writing – review & editing. NB: Formal analysis, Investigation, Visualization, Writing – review & editing. JC: Data curation, Funding acquisition, Methodology, Project administration, Writing – review & editing, Resources. BH: Data curation, Formal analysis, Methodology, Writing – review & editing, Resources. SS: Data curation, Formal analysis, Investigation, Methodology, Project administration, Writing – review & editing, Resources. RH: Conceptualization, Data curation, Formal analysis, Funding acquisition, Investigation, Project administration, Supervision, Validation, Visualization, Writing – original draft, Writing – review & editing, Resources.

## Supplementary material

Supplementary Data 1 The GFF3 file for the annotation of *T. tenax* sequence data from Benabdelkader et al. (2019) (zipped).

Supplementary Data 2 The GFF3 file for the annotation of *T. tenax* sequence data from Yang et al. (2022) (zipped).

Supplementary Data 3 The FASTA file with all 20,286 annotated proteins (integrated from the above two annotations) (flat text file).

Supplementary Table 1 *T. vaginalis* G3 look up table integrating and linking the 2007 and 2022 annotations (excel file).

Supplementary Table 2 *T. tenax* 20,286 locus tags for the protein coding genes, their annotation, protein length, TMHMM, SignalP and Phobius inferences and the protein sequence (excel table).

Supplementary Table 3 The CAZy annotation for both *T. tenax* and *T. vaginalis* G3_2007 (excel table).

Supplementary Table 4 The annotated peptidases for *T. tenax* (excel table).

Supplementary Table 5 The annotated BspA-like proteins for *T. tenax* (excel table).

Supplementary Table 6 The annotated Pmp-like proteins for *T. tenax* (excel table).

Supplementary Table 7 The annotated SAPLIP proteins for *T. tenax* (excel table).

Supplementary Table 8 The annotated ESCRT subunits for complex I-IV for *T. tenax* (excel table).

## References

[B1] Afgan E. Baker D. Van den Beek M. Blankenberg D. Bouvier D. Cech M.. (2016). The Galaxy platform for accessible, reproducible and collaborative biomedical analyses: 2016 update. Nucleic Acids Res. 44, W3–W10. 10.1093/nar/gkw343 27137889 PMC4987906

[B2] Alrefaei A. F. Low R. Hall N. Jardim R. Dávila A. Gerhold R.. (2019). Multilocus analysis resolves the European finch epidemic strain of *Trichomonas gallinae* and suggests introgression from divergent trichomonads. Genome Biol. Evol. 11, 2391–2402. 10.1093/gbe/evz164 31364699 PMC6735722

[B3] Altschul S. F. Gish W. Miller W. Myers E. W. Lipman D. J. (1990). Basic local alignment search tool. J. Mol. Biol. 215, 403–410. 10.1016/S0022-2836(05)80360-2 2231712

[B4] Artuyants A. Campos T. L. Rai A. K. Johnson P. J. Dauros-Singorenko P. Phillips A.. (2020). Extracellular vesicles produced by the protozoan parasite *Trichomonas vaginalis* contain a preferential cargo of tRNA-derived small RNAs. Int. J. Parasitol. 50, 1145–1155. 10.1016/j.ijpara.2020.07.003 32822680

[B5] Arumapperuma T. Li J. Hornung B. Soler N. M. Goddard-Borger E. D. Terrapon N.. (2023). A subfamily classification to choreograph the diverse activities within glycoside hydrolase family 31. J. Biol. Chem. 299:103038. 10.1016/j.jbc.2023.103038 36806678 PMC10074150

[B6] Aspeborg H. Coutinho P. M. Wang Y. Brumer H. Henrissat B. (2012). Evolution, substrate specificity and subfamily classification of glycoside hydrolase family 5 (GH5). BMC Evol. Biol. 12, 1–16. 10.1186/1471-2148-12-186 22992189 PMC3526467

[B7] Aurrecoechea C. Brestelli J. Brunk B. P. Carlton J. M. Dommer J. Fischer S.. (2009). GiardiaDB and TrichDB: integrated genomic resources for the eukaryotic protist pathogens *Giardia lamblia* and *Trichomonas vaginalis*. Nucleic Acids Res. 37, D526–D530. 10.1093/nar/gkn631 18824479 PMC2686445

[B8] Baker J. L. Mark Welch J. L. Kauffman K. M. McLean J. S. He X. (2024). The oral microbiome: diversity, biogeography and human health. Nat. Rev. Microbiol. 22, 89–104. 10.1038/s41579-023-00963-6 37700024 PMC11084736

[B9] Barnett M. J. Pinheiro J. Keown J. R. Biboy J. Gray J. Lucinescu I. W.. (2023). NlpC/P60 peptidoglycan hydrolases of *Trichomonas vaginalis* have complementary activities that empower the protozoan to control host-protective lactobacilli. PLoS Pathog. 19:e1011563. 10.1371/journal.ppat.1011563 37585473 PMC10461829

[B10] Barrett A. J. Rawlings N. D. (2001). Evolutionary lines of cysteine peptidases. Biol. Chem. 382, 727–734. 10.1515/bchm.2001.382.5.727 11517925

[B11] Benabdelkader S. Andreani J. Gillet A. Terrer E. Pignoly M. Chaudet H.. (2019). Specific clones of *Trichomonas tenax* are associated with periodontitis. PLoS ONE 14:e0213338. 10.1371/journal.pone.0213338 30856220 PMC6411126

[B12] Bisson C. Dridi S. M. Machouart M. (2019). Assessment of the role of *Trichomonas tenax* in the etiopathogenesis of human periodontitis: a systematic review. PLoS ONE 14:e0226266. 10.1371/journal.pone.0226266 31846467 PMC6917263

[B13] Briliute J. Urbanowicz P. A. Luis A. S. Basl,é A. Paterson N. Rebello O.. (2019). Complex N-glycan breakdown by gut *Bacteroides* involves an extensive enzymatic apparatus encoded by multiple co-regulated genetic loci. Nature Microbiol. 4, 1571–1581. 10.1038/s41564-019-0466-x 31160824 PMC7617214

[B14] Brosh-Nissimov T. Hindiyeh M. Azar R. Smollan G. Belausov N. Mandelboim M.. (2019). A false-positive *Trichomonas vaginalis* result due to *Trichomonas tenax* presence in clinical specimens may reveal a possible *T. tenax* urogenital infection. Clini. Microbiol. Infect. 25, 123–124. 10.1016/j.cmi.2018.09.011 30267929

[B15] Buchfink B. Reuter K. Drost H. G. (2021). Sensitive protein alignments at tree-of-life scale using DIAMOND. Nat. Methods 18, 366–368. 10.1038/s41592-021-01101-x 33828273 PMC8026399

[B16] Burge C. Karlin S. (1997). Prediction of complete gene structures in human genomic DNA. J. Mol. Biol. 268, 78–94. 10.1006/jmbi.1997.0951 9149143

[B17] Camacho C. Coulouris G. Avagyan V. Ma N. Papadopoulos J. Bealer K.. (2009). BLAST+: architecture and applications. BMC Bioinformat. 10, 1–9. 10.1186/1471-2105-10-421 20003500 PMC2803857

[B18] Carlton J. M. Hirt R. P. Silva J. C. Delcher A. L. Schatz M. Zhao Q.. (2007). Draft genome sequence of the sexually transmitted pathogen *Trichomonas vaginalis*. Science 315, 207–212. 10.1126/science.1132894 17218520 PMC2080659

[B19] Carlton J. M. Malik S. B. Sullivan S. A. Sicheritz-Pontén T. Tang P. Hirt R. P. (2010). “The genome of *Trichomonas vaginalis*,” in Anaerobic Parasitic Protozoa: Genomics and Molecular Biology. Wymondham: Caister Academic Press, 45–80.

[B20] Cepicka I. Hampl V. Kulda J. (2010). Critical taxonomic revision of parabasalids with description of one new genus and three new species. Protist 161, 400–433. 10.1016/j.protis.2009.11.005 20093080

[B21] Cóceres V. M. Alonso A. M. Nievas Y. R. Midlej V. Frontera L. Benchimol M.. (2015). The C-terminal tail of tetraspanin proteins regulates their intracellular distribution in the parasite *Trichomonas vaginalis*. Cell. Microbiol. 17, 1217–1229. 10.1111/cmi.12431 25703821

[B22] de Miguel N. Lustig G. Twu O. Chattopadhyay A. Wohlschlegel J. A. Johnson P. J. (2010). Proteome analysis of the surface of Trichomonas vaginalis reveals novel proteins and strain-dependent differential expression. Mol. Cellular Proteom. 9, 1554–1566. 10.1074/mcp.M000022-MCP201 20467041 PMC2938091

[B23] Diaz N. Lico C. Capodicasa C. Baschieri S. Dess,ì D. Benvenuto E.. (2020). Production and functional characterization of a recombinant predicted pore-forming protein (TVSAPLIP12) of *Trichomonas vaginalis* in *Nicotiana benthamiana* Plants. Front. Cell. Infect. Microbiol. 10:581066. 10.3389/fcimb.2020.581066 33117734 PMC7561387

[B24] Drula E. Garron M. L. Dogan S. Lombard V. Henrissat B. Terrapon N. (2022). The carbohydrate-active enzyme database: functions and literature. Nucleic Acids Res. 50, D571–D577. 10.1093/nar/gkab1045 34850161 PMC8728194

[B25] Emms D. M. Kelly S. (2015). OrthoFinder: solving fundamental biases in whole genome comparisons dramatically improves orthogroup inference accuracy. Genome Biol. 16:157. 10.1186/s13059-015-0721-2 26243257 PMC4531804

[B26] Eslahi A. V. Olfatifar M. Abdoli A. Houshmand E. Johkool M. G. Zarabadipour M.. (2021). The neglected role of *Trichomonas tenax* in oral diseases: a systematic review and meta-analysis. Acta Parasitologica 77, 715–732. 10.1007/s11686-021-00340-4 33595770

[B27] Feller L. Altini M. Khammissa R. A. G. Chandran R. Bouckaert M. Lemmer J. (2013). Oral mucosal immunity. Oral Surg. Oral Med. Oral Pathol. Oral Radiol. 116, 576–583. 10.1016/j.oooo.2013.07.013 24119522

[B28] Fichorova R. N. Buck O. R. Yamamoto H. S. Fashemi T. Dawood H. Y. Fashemi B.. (2013). The villain team-up or how *Trichomonas vaginalis* and bacterial vaginosis alter innate immunity in concert. Sex. Transm. Infect. 89, 460–466. 10.1136/sextrans-2013-051052 23903808 PMC3746192

[B29] Figueroa-Angulo E. E. Rendón-Gandarilla F. J. Puente-Rivera J. Calla-Choque J. S. Cárdenas-Guerra R. E. Ortega-López J.. (2012). The effects of environmental factors on the virulence of *Trichomonas vaginalis*. Microb. Infect. 14, 1411–1427. 10.1016/j.micinf.2012.09.004 23022315

[B30] Garcia M. R. Patel M. V. Shen Z. Fahey J. V. Biswas N. Mestecky J.. (2015). Mucosal immunity in the human female reproductive tract. Mucosal Immunol. 2015, 2097–2124. 10.1016/B978-0-12-415847-4.00108-7

[B31] Garron M. L. Henrissat B. (2019). The continuing expansion of CAZymes and their families. Curr. Opin. Chem. Biol. 53, 82–87. 10.1016/j.cbpa.2019.08.004 31550558

[B32] Haas B. J. Delcher A. L. Mount S. M. Wortman J. R. Smith Jr R. K. Hannick L. I.. (2003). Improving the Arabidopsis genome annotation using maximal transcript alignment assemblies. Nucleic Acids Res. 31, 5654–5666. 10.1093/nar/gkg770 14500829 PMC206470

[B33] Haas B. J. Salzberg S. L. Zhu W. Pertea M. Allen J. E. Orvis J.. (2008). Automated eukaryotic gene structure annotation using EVidenceModeler and the program to assemble spliced alignments. Genome Biol. 9:r7. 10.1186/gb-2008-9-1-r7 18190707 PMC2395244

[B34] Halary S. McInerney J. O. Lopez P. Bapteste E. (2013). EGN: a wizard for construction of gene and genome similarity networks. BMC Evol. Biol. 13, 1–9. 10.1186/1471-2148-13-146 23841456 PMC3727994

[B35] Hallgren J. Tsirigos K. D. Pedersen M. D. Almagro Armenteros J. J. Marcatili P. Nielsen H.. (2022). DeepTMHMM predicts alpha and beta transmembrane proteins using deep neural networks. BioRxiv [preprint]BioRxiv 2022–04. 10.1101/2022.04.08.487609

[B36] Handrich M. R. Garg S. G. Sommerville E. W. Hirt R. P. Gould S. B. (2019). Characterization of the BspA and Pmp protein family of trichomonads. Parasites Vectors 12:406. 10.1186/s13071-019-3660-z 31426868 PMC6701047

[B37] Hansson G. C. (2020). Mucins and the microbiome. Annu. Rev. Biochem. 89, 769–793. 10.1146/annurev-biochem-011520-105053 32243763 PMC8442341

[B38] Hernández-Romano P. Hernández R. Arroyo R. Alderete J. F. Lopez-Villasenor I. (2010). Identification and characterization of a surface-associated, subtilisin-like serine protease in *Trichomonas vaginalis*. Parasitology 137, 1621–1635. 10.1017/S003118201000051X 20602853

[B39] Hirt R. P. (2013). *Trichomonas vaginalis* virulence factors: an integrative overview. Sex. Transm. Infect. 89, 439–443. 10.1136/sextrans-2013-051105 23694938 PMC3749517

[B40] Hirt R. P. de Miguel N. Nakjang S. Dessi D. Liu Y. C. Diaz N.. (2011). *Trichomonas vaginalis* pathobiology: new insights from the genome sequence. Adv. Parasitol. 77, 87–140. 10.1016/B978-0-12-391429-3.00006-X 22137583

[B41] Honigberg B. M. (1990). “Trichomonads found outside the urogenital tract of humans,” in Trichomonads Parasitic in Humans (New York, NY: Springer), 342–393.

[B42] Huerta-Cepas J. Forslund K. Coelho L. P. Szklarczyk D. Jensen L. J. Von Mering C.. (2017). Fast genome-wide functional annotation through orthology assignment by eggNOG-mapper. Mol. Biol. Evol. 34, 2115–2122. 10.1093/molbev/msx148 28460117 PMC5850834

[B43] Huerta-Cepas J. Szklarczyk D. Forslund K. Cook H. Heller D. Walter M. C.. (2016). eggNOG 4.5: a hierarchical orthology framework with improved functional annotations for eukaryotic, prokaryotic and viral sequences. Nucleic Acids Res. 44, D286–D293. 10.1093/nar/gkv1248 26582926 PMC4702882

[B44] Humann J. L. Lee T. Ficklin S. P. Cheng C. H. Hough H. Jung S.. (2019). “GenSAS v6. 0: a web-based platform for structural and functional annotation of model and non-model organisms,” in Plant and Animal Genome XXVII Conference (January 12–16, 2019) (PAG).

[B45] Irazoki O. Hernandez S. B. Cava F. (2019). Peptidoglycan muropeptides: release, perception, and functions as signaling molecules. Front. Microbiol. 10:429805. 10.3389/fmicb.2019.00500 30984120 PMC6448482

[B46] Jones P. Binns D. Chang H. Y. Fraser M. Li W. McAnulla C.. (2014). InterProScan 5: genome-scale protein function classification. Bioinformatics 30, 1236–1240. 10.1093/bioinformatics/btu031 24451626 PMC3998142

[B47] Käll L. Krogh A. Sonnhammer E. L. (2007). Advantages of combined transmembrane topology and signal peptide prediction-the Phobius web server. Nucleic Acids Res. 35(Suppl_2), W429-W432. 10.1093/nar/gkm256 17483518 PMC1933244

[B48] Kanehisa M. (2002). “The KEGG database,” in ‘*In Silico' Simulation of Biological Processes: Novartis Foundation Symposium, Vol. 247* (Chichester: John Wiley & Sons, Ltd.), 91–103.

[B49] Kanehisa M. Sato Y. Morishima K. (2016). BlastKOALA and GhostKOALA: KEGG tools for functional characterization of genome and metagenome sequences. J. Mol. Biol. 428, 726–731. 10.1016/j.jmb.2015.11.006 26585406

[B50] Kellerová P. Tachezy J. (2017). Zoonotic Trichomonas tenax and a new trichomonad species, *Trichomonas brixi* n. sp., from the oral cavities of dogs and cats. Int. J. Parasitol. 47, 247–255. 10.1016/j.ijpara.2016.12.006 28238869

[B51] Kelly S. Maini P. K. (2013). DendroBLAST: approximate phylogenetic trees in the absence of multiple sequence alignments. PLoS ONE 8:e58537. 10.1371/journal.pone.0058537 23554899 PMC3598851

[B52] Kent W. J. (2002). BLAT—the BLAST-like alignment tool. Genome Res. 12, 656–664. 10.1101/gr.229202 11932250 PMC187518

[B53] Kim D. Langmead B. Salzberg S. L. (2015). HISAT: a fast spliced aligner with low memory requirements. Nat. Methods 12, 357–360. 10.1038/nmeth.3317 25751142 PMC4655817

[B54] Kim D. Pertea G. Trapnell C. Pimentel H. Kelley R. Salzberg S. L. (2013). TopHat2: accurate alignment of transcriptomes in the presence of insertions, deletions and gene fusions. Genome Biol. 14:r36. 10.1186/gb-2013-14-4-r36 23618408 PMC4053844

[B55] Korf I. (2004). Gene finding in novel genomes. BMC Bioinformatics 5:59. 10.1186/1471-2105-5-59 15144565 PMC421630

[B56] Kriventseva E. V. Rahman N. Espinosa O. Zdobnov E. M. (2007). OrthoDB: the hierarchical catalog of eukaryotic orthologs. Nucleic Acids Res. 36(Suppl_1), D271-D275. 10.1093/nar/gkm845 17947323 PMC2238902

[B57] Labourel A. Parrou J. L. Deraison C. Mercier-Bonin M. Lajus S. Potocki-Veronese G. (2023). O-Mucin-degrading carbohydrate-active enzymes and their possible implication in inflammatory bowel diseases. Essays Biochem. 67:331. 10.1042/EBC20220153 36912232 PMC10154620

[B58] Letunic I. Bork P. (2018). 20 years of the SMART protein domain annotation resource. Nucleic Acids Res. 46, D493–D496. 10.1093/nar/gkx922 29040681 PMC5753352

[B59] Leung K. F. Dacks J. B. Field M. C. (2008). Evolution of the multivesicular body ESCRT machinery; retention across the eukaryotic lineage. Traffic 9, 1698–1716. 10.1111/j.1600-0854.2008.00797.x 18637903

[B60] Lombard V. Golaconda Ramulu H. Drula E. Coutinho P. M. Henrissat B. (2014). The carbohydrate-active enzymes database (CAZy) in 2013. Nucleic Acids Res. 42, D490–D495. 10.1093/nar/gkt1178 24270786 PMC3965031

[B61] Lomsadze A. Ter-Hovhannisyan V. Chernoff Y. O. Borodovsky M. (2005). Gene identification in novel eukaryotic genomes by self-training algorithm. Nucleic Acids Res. 33, 6494–6506. 10.1093/nar/gki937 16314312 PMC1298918

[B62] Ma L. Meng Q. Cheng W. Sung Y. Tang P. Hu S.. (2011). Involvement of the GP63 protease in infection of Trichomonas vaginalis. Parasitol. Res. 109, 71–79. 10.1007/s00436-010-2222-2 21221643

[B63] Manni M. Berkeley M. R. Seppey M. Simão F. A. Zdobnov E. M. (2021). BUSCO update: novel and streamlined workflows along with broader and deeper phylogenetic coverage for scoring of eukaryotic, prokaryotic, and viral genomes. Mol. Biol. Evol. 38, 4647–4654. 10.1093/molbev/msab199 34320186 PMC8476166

[B64] Margarita V. Bailey N. P. Rappelli P. Diaz N. Dess,ì D. Fettweis J. M.. (2022). Two different species of Mycoplasma endosymbionts can influence *Trichomonas vaginalis* pathophysiology. MBio 13, e00918–e00922. 10.1128/mbio.00918-22 35608298 PMC9239101

[B65] Margarita V. Congiargiu A. Diaz N. Fiori P. L. Rappelli P. (2023). *Mycoplasma hominis* and *Candidatus Mycoplasma girerdii* in *Trichomonas vaginalis*: peaceful cohabitants or contentious roommates? Pathogens 12:1083. 10.3390/pathogens12091083 37764891 PMC10535475

[B66] Maritz J. M. Land K. M. Carlton J. M. Hirt R. P. (2014). What is the importance of zoonotic trichomonads for human health? Trends Parasitol. 30, 333–341. 10.1016/j.pt.2014.05.005 24951156 PMC7106558

[B67] Martin-Garcia D. F. Sallam M. Garcia G. Santi-Rocca J. (2022). Parasites in periodontal health and disease: a systematic review and meta-analysis. Periodontitis: Adv. Exp. Res. 2022, 95–111. 10.1007/978-3-030-96881-6_5 35612794

[B68] Marty M. Lemaitre M. Kemoun P. Morrier J. J. Monsarrat P. (2017). *Trichomonas tenax* and periodontal diseases: a concise review. Parasitology 144, 1417–1425. 10.1017/S0031182017000701 28583214

[B69] Matthew M. A. Yang N. Ketzis J. Mukaratirwa S. Yao C. (2023). *Trichomonas tenax*: a neglected protozoan infection in the oral cavities of humans and dogs—a scoping review. Trop. Med. Infect. Dis. 8:60. 10.3390/tropicalmed8010060 36668967 PMC9863487

[B70] Mercer F. Johnson P. J. (2018). *Trichomonas vaginalis*: pathogenesis, symbiont interactions, and host cell immune responses. Trends Parasitol. 34, 683–693. 10.1016/j.pt.2018.05.006 30056833 PMC11132421

[B71] Molgora B. M. Mukherjee S. K. Baumel-Alterzon S. Santiago F. M. Muratore K. A. Sisk Jr A. E.. (2023). *Trichomonas vaginalis* adherence phenotypes and extracellular vesicles impact parasite survival in a novel in vivo model of pathogenesis. PLoS Negl. Trop. Dis. 17, e0011693. 10.1371/journal.pntd.0011693 37871037 PMC10621976

[B72] Nakjang S. Ndeh D. A. Wipat A. Bolam D. N. Hirt R. P. (2012). A novel extracellular metallopeptidase domain shared by animal host-associated mutualistic and pathogenic microbes. PLoS ONE 7:e30287. 10.1371/journal.pone.0030287 22299034 PMC3267712

[B73] Nishimura D. (2000). RepeatMasker. Biotech. Softw. Internet Rep. 1, 36–39. 10.1089/152791600319259

[B74] Noël C. J. Diaz N. Sicheritz-Ponten T. Safarikova L. Tachezy J. Tang P.. (2010). *Trichomonas vaginalis* vast BspA-like gene family: evidence for functional diversity from structural organisation and transcriptomics. BMC Genomics 11:99. 10.1186/1471-2164-11-99 20144183 PMC2843621

[B75] Ong S. C. Luo H. W. Cheng W. H. Ku F. M. Tsai C. Y. Huang P. J.. (2024). The core exosome proteome of *Trichomonas vaginalis*. J. Microbiol. Immunol. Infect. 57, 246–256. 10.1016/j.jmii.2024.02.003 38383245

[B76] Petersen T. N. Brunak S. Von Heijne G. Nielsen H. (2011). SignalP 4.0: discriminating signal peptides from transmembrane regions. Nat. Methods 8, 785–786. 10.1038/nmeth.1701 21959131

[B77] Rai A. K. Johnson P. J. (2019). *Trichomonas vaginalis* extracellular vesicles are internalized by host cells using proteoglycans and caveolin-dependent endocytosis. Proc. Nat. Acad. Sci. 116, 21354–21360. 10.1073/pnas.1912356116 31601738 PMC6815132

[B78] Ribeiro L. C. Santos C. Benchimol M. (2015). Is *Trichomonas tenax* a parasite or a commensal? Protist 166, 196–210. 10.1016/j.protis.2015.02.002 25835639

[B79] Riestra A. M. de Miguel N. Dessi D. Simoes-Barbosa A. Mercer F. K. (2022). “*Trichomonas vaginalis*: lifestyle, cellular biology, and molecular mechanisms of pathogenesis,” in Lifecycles of Pathogenic Protists in Humans (Cham: Springer International Publishing), 541-617.

[B80] Salas N. Pedreros M. B. dos Santos Melo T. Maguire V. G. Sha J. Wohlschlegel J. A.. (2023). Role of cytoneme structures and extracellular vesicles in *Trichomonas vaginalis* parasite-parasite communication. Elife 12:e86067. 10.7554/eLife.86067.sa2 37129369 PMC10270686

[B81] Salzberg S. L. Pertea M. Delcher A. L. Gardner M. J. Tettelin H. (1999). Interpolated Markov models for eukaryotic gene finding. Genomics 59, 24–31. 10.1006/geno.1999.5854 10395796

[B82] Santi-Rocca J. (2020). “The Protozoome of the Periodontal Sulcus: from health to disease,” in Eukaryome Impact on Human Intestine Homeostasis and Mucosal Immunology: Overview of the First Eukaryome Congress at Institut Pasteur. (Paris: Springer International Publishing), 113–131.

[B83] Shannon P. Markiel A. Ozier O. Baliga N. S. Wang J. T. Ramage D.. (2003). Cytoscape: a software environment for integrated models of biomolecular interaction networks. Genome Res. 13, 2498–2504. 10.1101/gr.1239303 14597658 PMC403769

[B84] Sharma A. (2010). Virulence mechanisms of *Tannerella forsythia*. Periodontol. 54:106. 10.1111/j.1600-0757.2009.00332.x 20712636 PMC2934765

[B85] Smit A. Hubley R. (2008). RepeatModeler Open-1.0. Repeat Masker. Available online at: http://www.repeatmasker.org

[B86] Stanke M. Steinkamp R. Waack S. Morgenstern B. (2004). AUGUSTUS: a web server for gene finding in eukaryotes. Nucleic Acids Res. 32(Suppl_2), W309-W312. 10.1093/nar/gkh379 15215400 PMC441517

[B87] Sun J. Lu F. Luo Y. Bie L. Xu L. Wang Y. (2023). OrthoVenn3: an integrated platform for exploring and visualizing orthologous data across genomes. Nucleic Acids Res. 51, W397-W403. 10.1093/nar/gkad313 37114999 PMC10320085

[B88] Törönen P. Medlar A. Holm L. (2018). PANNZER2: a rapid functional annotation web server. Nucleic Acids Res. 46, W84–W88. 10.1093/nar/gky350 29741643 PMC6031051

[B89] Twu O. de Miguel N. Lustig G. Stevens G. C. Vashisht A. A. Wohlschlegel J. A.. (2013). *Trichomonas vaginalis* exosomes deliver cargo to host cells and mediate host: parasite interactions. PLoS Pathog. 9:e1003482. 10.1371/journal.ppat.1003482 23853596 PMC3708881

[B90] Viborg A. H. Terrapon N. Lombard V. Michel G. Czjzek M. Henrissat B.. (2019). A subfamily roadmap of the evolutionarily diverse glycoside hydrolase family 16 (GH16). J. Biol. Chem. 294, 15973–15986. 10.1074/jbc.RA119.010619 31501245 PMC6827312

[B91] Wardman J. F. Bains R. K. Rahfeld P. Withers S. G. (2022). Carbohydrate-active enzymes (CAZymes) in the gut microbiome. Nat. Rev. Microbiol. 20, 542–556. 10.1038/s41579-022-00712-1 35347288

[B92] Yang N. Christie J. Keen H. L. Matthew M. A. Yao C. (2022). Draft genome sequence of *Trichomonas tenax* strain Hs-4: NIH. Microbiol. Res. Announcem. 11, e00157–e00122. 10.1128/mra.00157-22 35861552 PMC9302087

[B93] Zubáčová Z. Cimburek Z. Tachezy J. (2008). Comparative analysis of trichomonad genome sizes and karyotypes. Mol. Biochem. Parasitol. 161, 49–54. 10.1016/j.molbiopara.2008.06.004 18606195

